# Editorial: Hormonal control of plant stress responses: Brassinosteroids and gibberellin

**DOI:** 10.3389/fpls.2023.1131398

**Published:** 2023-01-18

**Authors:** Dawei Zhang

**Affiliations:** Ministry of Education Key Laboratory for Bio-Resource and Eco-Environment, College of Life Sciences, State Key Laboratory of Hydraulics and Mountain River Engineering, Sichuan University, Chengdu, China

**Keywords:** plant hormones, Brassinosteroids, gibberellin, abiotic and biotic stresses, regulatory mechanisms

As sessile organisms, plants have evolved sophisticated regulatory networks to adjust their growth patterns and developmental programs accordingly in order to better respond and adapt to ever-changing environments. Plant hormones serve as important signaling molecules to coordinate the growth and development responses to the external stimuli, as well as mediate plant acclimation to numerous adverse conditions. Among phytohormones, Brassinosteroids (BRs) and gibberellin (GA) play essential roles in normal growth and development and in responses to biotic/abiotic stresses, how they coordinate plant development and responses to the environment and the relationships of BRs or GA with other signaling pathways require further investigation. This Research Topic reflects the latest advances in the role of BRs or GA in regulating plant abiotic/biotic stress responses, focusing on physiological, cellular, and biochemical effects, as well as underlying genetic determination and molecular control. The research Topic includes five original research articles and one review article.

Brassinosteroids (BRs) are a group of steroid hormones that play essential roles in plant growth, development and stress response. Over the past several decades, BR biosynthesis and signaling pathway have been clearly established (Choudhary et al., 2012). Subsequently, increasing research has focused on the roles of BRs in coordinating plant growth and stress responses. Especially, resent studies of Yanhai Yin’s group have systematically revealed how plants coordinate growth and drought tolerance through fine-tuning BR signaling pathway (Nolan et al., 2020). BIN2 and BES1/BZR1, two BR signaling components, have emerged as key nodes in promoting and antagonizing drought responses and played important roles in mediates the crosstalk of BR and drought response (Nolan et al., 2017). In this topic, a study by Tang et al. implicated that BR biosynthesis pathway was involved in *Mesona chinensis Benth* (MCB) response to drought stress, indicating that the roles of BRs in drought response are conserved in multiple plant species. Additionally, an original research article by Ren et al., revealed a novel regulatory pathway mediating the crosstalk between BR signaling and plant heat response. BRs signaling were initially reported to protect plants from heat stress *via* reducing the heat damage when applied externally, while the underlying mechanism is still unclear (Dhaubhadel et al., 2002; Divi et al., 2016). In this study, the authors systematically analyzed the thermotolerance of BR biosynthesis or BR signaling loss-of-function or gain-of-function mutants of Arabidopsis and identified BIN2 as a key node linking the BR signaling and plant heat stress response. Interesting, this study found heat stress promoted BIN2 accumulation, which then accelerated the life cycle of plants under heat stress conditions by promoting early flower, while BIN2 negative regulated plants thermotolerance. Therefore, this study discovered a novel function of BIN2 in regulatory of plant acclimation to heat stress in a tissue-specific manner and uncovered the mechanism of BR signaling promoting thermotolerance.

In addition to stress conditions, nutritional deficiency also acts as an adverse environment which profoundly affects plants growth, development and the productivity of crops. BRs are involved in regulation of the “forging responses” of plant lateral roots in response to nutritional deficiency. Two recent studies identified BR biosynthesis gene DWF4 and signaling component BSK3 as essential regulators in Arabidopsis mediating the BR-induced lateral root elongation in response to low nitrogen, but the downstream regulatory mechanism is unknown. In this topic, Chai et al. expanded our knowledge of the molecular mechanism by which BR signaling adjusts the “forging response” of plant lateral roots under low nitrogen conditions. The article identified an interactor of BES1, LBD37, which is a well-known negative regulator of N availability signals. BES1 suppressed LBD37 at both transcription and protein levels, thereby upsetting the inhibitory effects of LBD37 on nitrate transporter and reductase, such as *NRT1.1*, *NRT2.1* and *ANR1* and allowing the elongation of plants lateral roots under low nitrogen conditions.

Additionally, Xiong et al. implicated a novel mechanism of BRs in regulation of biotic stress in *Arabidopsis*. Several BR synthesis or BR signaling deficient mutants exhibited susceptibility to *Pst*DC3000 infection. Interestingly, *GLUCAN SYNTHASE-LIKE 8 (GSL8)*, a key synthase involved in callose biosynthesis, was identified as a direct target of BES1. BRs induced pathogen resistance was dependent on BES1-GSL8 module, as Brassinolide (BL) treatment-promoted *Pst*DC3000 resistance was impaired in *gsl8-1* knock-out mutant. This article presented the growth-promoted regulator BES1 also as a pathogen resistance-related gene, which could be a valuable target for breeding pathogen-resistant crops.

Other articles in this collection evaluated the roles of another growth-promoting phytohormone gibberellin in rice salt tolerance and agronomic traits of horticultural plants. A research article by Farooq et al. showed the impact of NaCl treatment along with exogenous GA3 in different rice cultivars. In these rice cultivars, including two famous rice cultivars, Cheongcheong and Nagdong, a salt-sensitive IR28, and a salt-tolerant Pokkali, 120mM NaCl treatment could significantly reduce their seed germination rates, seedling growth and GA content *via* regulating the expression of genes related to GA production and metabolism. However, exogenous 50 μM GA3 treatment could alleviate the effects of salt stress on salt-tolerant Pokkali and Nagdong cultivars, while salt-sensitive IR28 and Cheongcheong cultivars should be treated with 100 μM GA3. Another review article by Zhang et al. described the role of GA in regulating plant stature, axillary meristem outgrowth, compound leaf development, flowering time, and parthenocarpy in horticultural plants. The article described the functions and regulation of the components of GA biosynthesis and signaling pathway identified in horticultural plants and discussed how GAs regulates vegetative and reproduction growth of horticultural plants. It is of particular importance to elucidate the genetic and molecular functions of GA in horticultural plant, which may promote the development of novel horticultural plants with high quality and quantity *via* manipulating GA contents and GA signaling.

This Research Topic provides important advances in the role of BRs or GA in regulating plant abiotic/biotic stress responses and highlights the physiological, cellular, and biochemical effects, as well as underlying genetic determination and molecular controls of these two phytohormones. However, much progress of this topic were made in model plant Arabidopsis, the functions of BRs and GA in crops should be get more attention in the future.

## Author contributions

The author confirms being the sole contributor of this work and has approved it for publication.
